# Tuning the Electronic, Ion Transport, and Stability Properties of Li-rich Manganese-based Oxide Materials with Oxide Perovskite Coatings: A First-Principles Computational Study

**DOI:** 10.1021/acsami.2c07560

**Published:** 2022-08-05

**Authors:** Zizhen Zhou, Dewei Chu, Bo Gao, Toshiyuki Momma, Yoshitaka Tateyama, Claudio Cazorla

**Affiliations:** † School of Materials Science and Engineering, UNSW Australia, Sydney, NSW 2052, Australia; ‡ Graduate School of Advanced Science and Engineering, Waseda University, 3-4-1, Okubo, Shinjuku-ku, Tokyo 169-8555, Japan; § Center for Green Research on Energy and Environmental Materials (GREEN) and International Center for Materials Nanoarchitectonics (MANA), National Institute for Materials Science (NIMS), 1-1 Namiki, Tsukuba, Ibaraki 305-0044, Japan; ∥ Departament de Física, Universitat Politècnica de Catalunya, Campus Nord B4-B5, E-08034 Barcelona, Spain

**Keywords:** lithium battery, Li-rich manganese-based cathode, ionic diffusion, oxide perovskite, density functional theory, interface modeling

## Abstract

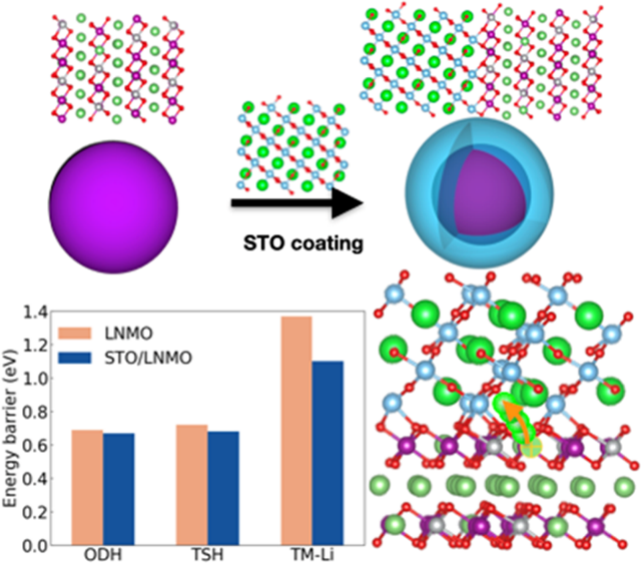

Lithium-rich manganese-based oxides (LRMO) are regarded as promising cathode materials for powering electric applications due to their high capacity (250 mAh g^–1^) and energy density (∼900 Wh kg^–1^). However, poor cycle stability and capacity fading have impeded the commercialization of this family of materials as battery components. Surface modification based on coating has proven successful in mitigating some of these problems, but a microscopic understanding of how such improvements are attained is still lacking, thus impeding systematic and rational design of LRMO-based cathodes. In this work, first-principles density functional theory (DFT) calculations are carried out to fill out such a knowledge gap and to propose a promising LRMO-coating material. It is found that SrTiO_3_ (STO), an archetypal and highly stable oxide perovskite, represents an excellent coating material for Li_1.2_Ni_0.2_Mn_0.6_O_2_ (LNMO), a prototypical member of the LRMO family. An accomplished atomistic model is constructed to theoretically estimate the structural, electronic, oxygen vacancy formation energy, and lithium-transport properties of the LNMO/STO interface system, thus providing insightful comparisons with the two integrating bulk materials. It is found that (i) electronic transport in the LNMO cathode is enhanced due to partial closure of the LNMO band gap (∼0.4 eV) and (ii) the lithium ions can easily diffuse near the LNMO/STO interface and within STO due to the small size of the involved ion-hopping energy barriers. Furthermore, the formation energy of oxygen vacancies notably increases close to the LNMO/STO interface, thus indicating a reduction in oxygen loss at the cathode surface and a potential inhibition of undesirable structural phase transitions. This theoretical work therefore opens up new routes for the practical improvement of cost-affordable lithium-rich cathode materials based on highly stable oxide perovskite coatings.

## 1 Introduction

Li-ion batteries (LIBs) have powered portable electronic devices for about three decades.^1,2^ Recently, the use of LIBs has been extended to electric vehicles, which, in order to make positive commercial and environmental impacts, require high autonomy mileage and cost-affordability.^3^ However, traditional LIBs based on cathode materials like LiCoO_2_ typically deliver output capacities of <200 mAh g^–1^ and energy densities of <220 Wh kg^–1^,^4^ which fail to fulfill the automobile demand of >350 Wh kg^–1^.^5,6^ Thus, considerable research has been recently conducted to identify and design improved cathode materials.

Li-rich manganese based oxides (LRMO) with general formula *x*Li_2_MnO_3_·(1 – *x*)LiMO_2_ (1 < *x* ≤ 1/3; M = Ni, Co, Mn) are regarded as promising candidates to ameliorate the gap between LIB supply and demand owing to their great energy capacities (typically >250 mAh g^–1^) and energy densities (∼900 Wh kg^–1^).^5^ However, LRMOs present quite limiting practical issues like large first-cycle irreversible capacity loss, unsatisfactory cycle performance, and low rate capability.^7^ The main causes of these practical issues include an unwanted transition toward a layered-spinel phase and oxygen-redox evolution reactions occurring during electrochemical cycling.^8^ Consequently, the exploitation of LRMO in advanced electric vehicle and portable electronic devices remains significantly hindered.^6,9,10^

To overcome these shortcomings, extensive efforts have been made to enhance the electrochemical performance of LRMO cathode materials including doping,^11,12^ surface modification,^13−15^ and particle size control.^16,17^ Nanosizing has been employed to enhance the structural stability and electrochemical performance of LRMO; however, the accompanying material agglomeration (i.e., adhesion of nanoparticles) and triggering of electrolyte-induced side reactions due to increased surface energy and area^18−20^ have discouraged the use of this approach. Doping strategies, on the other hand, have been partially successful in stabilizing LRMO-based electrodes. For instance, Wang *et al.* have employed Mg as an additional dopant species to synthesize the compound Li[Li_0.2–2*x*_Mg_*x*_Co_0.13_Ni_0.13_Mn_0.54_]O_2_ (*x* = 0.02, 0.04, and 0.06), finding that *x* = 0.04 exhibits improved energy capacity and rate capability.^21^ Nonetheless, the chemical engineering associated with such doping strategies remains challenging and poorly understood, thus frustrating systematic and rational design of cathode materials.^22^

Surface modification via coating is considered a successful solution to protect electrodes without changing their bulk material structure. Thus far, various electrode-coating materials have been reported, including oxides (Co_3_O_4_,^23^ TiO_2_,^24^ CeO_2_^25^), phosphates (AlPO_4_,^26^ FePO_4_^27^), and graphite fluorides.^13^ Most of these coating materials act as physical barriers protecting the electrode from the hydrofluoric attacks caused by the acids contained in the electrolyte. Coating materials can also prevent unwanted layer-to-spinel phase transitions and promote electronic conductivity within the cathode, which in some cases may be desirable.^28,29^

In terms of rational design, ideal cathode-coating materials need to be protective to prevent possible cathode phase transitions, electrolyte decomposition, and the appearance of microcracks.^31^ Prospective coating materials should also avoid the rise of charge transfer resistance and conversely be able to improve the electrochemical performance of the electrodes. Interestingly, previous computational work has also shown that reducing electron transfer between the electrolyte and cathode may be a crucial practical aspect.^32^

Nevertheless, elimination of the oxygen that results from the side evolution reactions occurring within the coated cathodes, which causes poor LRMO electrochemical performance, remains a big challenge. In addition, the extent to which the coating material may affect the Li-ion diffusivity near the created interface and lead to unwanted reduction of the charge–discharge capacities is not well understood.^30^ Therefore, in order to design LIBs with enhanced cyclic stabilities and rate capabilities, it is crucial to improve our current understanding of LRMO/coating material interfaces at the structural, electronic, and Li-ion transport levels.

Here, we report a first-principles simulation study on the structural, electronic, Li-ion transport, and stability properties of the cathode material Li_1.2_Ni_0.2_Mn_0.6_O_2_ (LNMO) coated with SrTiO_3_ (STO)*.* LNMO is selected as a representative LRMO compound owing to its high economical affordability, hypotoxicity, large discharge capacity, and reasonable stability.^33^ Meanwhile, STO, an archetypal oxide perovskite, has been chosen based on its high thermodynamic stability, elemental abundance, non-toxicity, synthesis versatility, and structural compatibility with LNMO. Our first-principles density functional theory (DFT) calculations show that (i) STO coating layers can successfully accommodate Li ions as well as to enhance the Li ion diffusivity in the LNMO cathode, (ii) the formation energy of oxygen vacancies is significantly reduced at the LNMO/STO interface, which may prevent structure cathode degradation, and (iii) the density of electronic states at the LNMO/STO interface is enhanced as compared to that of bulk LNMO, thus promoting electronic transport inside the LNMO cathode. The conclusions presented here for a model LNMO/STO interface system are expected to be generalizable to other LRMO and perovskite-based materials; thus, the present work advances knowledge in the design of more stable and energy efficient LIBs based on cathode-coating techniques.

## 2 Computational Methods

Spin-polarized first-principles calculations based on density functional theory (DFT) were carried out.^34^ The PBE exchange-correlation energy functional^35^ was used as it is implemented in the VASP software.^36^ The projector-augmented wave method (PAW)^37^ was employed to represent the ionic cores by considering the following electronic states as valence: Sr 4*s*, 4*p*, and 5*s*; Ti 3*p*, 4*s*, and 3*d*; O 2*s* and 2*p*; Li 1*s*, 2*s*, and 2*p*; Mn 3*p*, 4*s*, and 3*d*; Ni 3*p*, 4*s*, and 3*d*. A “Hubbard-*U*” scheme^38^ was employed for a better treatment of the Ti, Mn, and Ni and 3*d* electric orbitals, with a selected *U* value of 2.0, 3.9, and 6.0 eV,^39−41^ respectively. An energy cutoff of 520 eV and a Monkhorst–Pack *k*-point grid of 2 × 2 × 1 for integrations within the Brillouin zone were used in the geometry optimizations, which led to total energies converged to within 1 meV per atom. Atomic relaxations were concluded when the forces in the atoms were all below 0.01 eV Å^–1^. The STO/LNMO supercell employed in our simulations contained a total of 384 atoms and was at all practical effects symmetric along the direction perpendicular to the two-material interface (Figure S1). Analysis of the macroscopic average electrostatic potential calculated in the STO/LNMO system along the same direction suggests correct convergence of our DFT results with respect to the slabs’ thickness (Figure S2).

**Figure 1:**
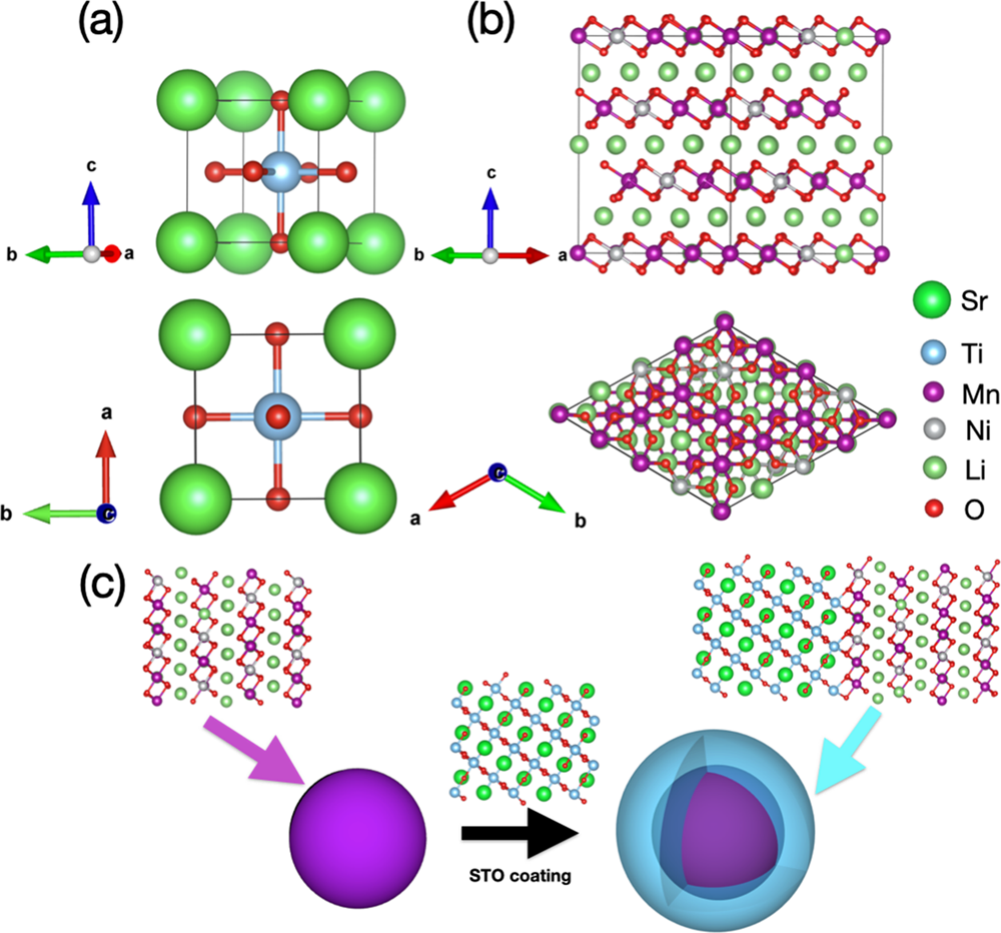
Atomic bulk structures of STO (a) and LNMO (b) as considered in this study. Sketch of the LNMO-coating procedure based on STO (c).

The bulk atomic structure of SrTiO_3_ (STO) and Li_1.2_Ni_0.2_Mn_0.6_O_2_ (LNMO) considered in this work are shown in Figure 1. STO adopts the typical paraelectric perovskite phase (cubic symmetry, space group *Pm*3̅*m*) and LNMO was built by replacing Mn atoms with Ni and Li ions in a 4 × 4 × 1 LiMnO_2_ (rhombohedral symmetry, space group *R*3̅*m*) supercell. Regarding the LNMO structure, different possible cationic arrangements were initially generated and ranked according to their electrostatic energy by using the Pymatgen package;^42^ subsequently, all of them were geometrically optimized with DFT methods. Figure S3 shows the top 30 configurations ranked by Pymatgen according to their electrostatic interactions and the corresponding DFT energies. The one with the lowest DFT energy [Figure S3, (1)] was employed in all the subsequent STO/LNMO calculations. The lattice constants of the relaxed bulk LNMO and bulk STO systems are shown in Table S1, and very good agreement was found with respect to the available experimental data.

By considering additional LNMO configurations, even lower-energy atomic arrangements could possibly be identified. Nevertheless, the general conclusions presented in this article are not expected to significantly depend on the very specific details of the selected LNMO configuration since in most cases the DFT calculations have been performed for different positions and as a function of the distance to the STO/LNMO interface; hence, local Mn-Ni-Li environment effects should be already averaged out in them.

Due to the presence of high chemical disorder in the LNMO system, in our DFT calculations, we initialized all the Mn and Ni ions (a total of 39) with parallel magnetic moments (i.e., ferromagnetic spin ordering) and all the Li and O ions (a total of 153) with null magnetic moments. Upon relaxation of the system toward the ground state, we found that most of the Mn ions conserved their parallel magnetic moments (∼93%), although few of them switched to anti-parallel (∼7%); regarding the Ni ions, they most exhibited anti-parallel spin ordering (i.e., half of the Ni ions exhibited “up” magnetic moments, and the other half exhibited “down” magnetic moments). As expected, most of the oxygen ions became partially magnetized, and their net magnetic moments were predominantly “down” (∼80%). Due to the highly variable atomic environment around the transition metal ions in LNMO, it was not possible to determine a regular magnetic ordering in the corresponding relaxed structure.

Ab initio nudged-elastic band (NEB) calculations^43^ were conducted to estimate the energy barrier (*E*_B_) of Li migration in bulk STO, bulk LNMO, and the STO/LNMO interface system. In this case, we used a 1 × 1 × 1 **Γ**-centered *k*-point grid for Brillouin zone sampling and an energy cutoff of 400 eV. The geometry optimizations were halted when the forces on the atoms were all smaller than 0.01 eV Å^–1^. Following previous computational studies on Li-ion diffusion in layered cathode materials, the standard PBE functional (i.e., without any *U*) was employed in our NEB calculations to avoid the mixing of the ionic migration barriers with a charge transfer barrier.^44,45^

For the calculation of vacancy formation energies, *E*_V_, we removed one single oxygen or lithium ion from the simulation supercell, which leads to defect concentrations of ∼0.4 and ∼ 1.6%, respectively. For the sake of computational affordability and in view of previous first-principles results reported on bulk STO,^41^ in the present study, all point defects have been assumed to be neutrally charged; the formation energy of vacancies, *E*_V_, therefore, was estimated by using the formula:
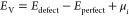
1where *E*_defect_ is the total energy of the system containing one O or Li vacancy, *E*_perfect_ is the total energy of the system without any defect, and μ_*i*_ is the chemical potential of the removed ion. μ_Li_ was estimated from the formation energy of bulk bcc Li and was found to be equal to −1.904 eV. μ_O_ was adopted from previous computational studies,^46,47^ in which a correction of 0.68 eV was considered to compensate for the usual PBE underestimation, thus being equal to μ_O_ = −4.26 eV. Since the types of vacancies considered in this study were charge-neutral, we excluded energy terms correcting for periodic charge-image spurious effects.^48^ Point defects other than oxygen and lithium vacancies have been neglected in this study due to obvious computational limitations.

Charge density distribution differences (CDD) were calculated to substantiate the redistribution of charges in the LNMO cathode upon STO coating, according to the formula
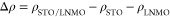
2where ρ_STO/LNMO_ is the total charge density of the LNMO/STO interface system, and ρ_STO_ and ρ_LNMO_ are the charge density of the isolated STO coating and LNMO cathode calculated separately.

In addition, the surface energy of the analyzed slabs, *E*_surf_, and the adhesion energy of the joint STO/LNMO system, *E*_adh_, were estimated with the formulas
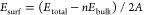
3
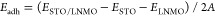
4where *E*_total_ represents the energy of the slab, *n* represents the number of formula units in the slab, *E*_bulk_ represents the bulk energy per formula unit, and *A* represents the surface of the slabs.

## 3 Results and Discussion

### 3.1 Structural and Electronic Properties of the STO/LNMO System

**Figure 2:**
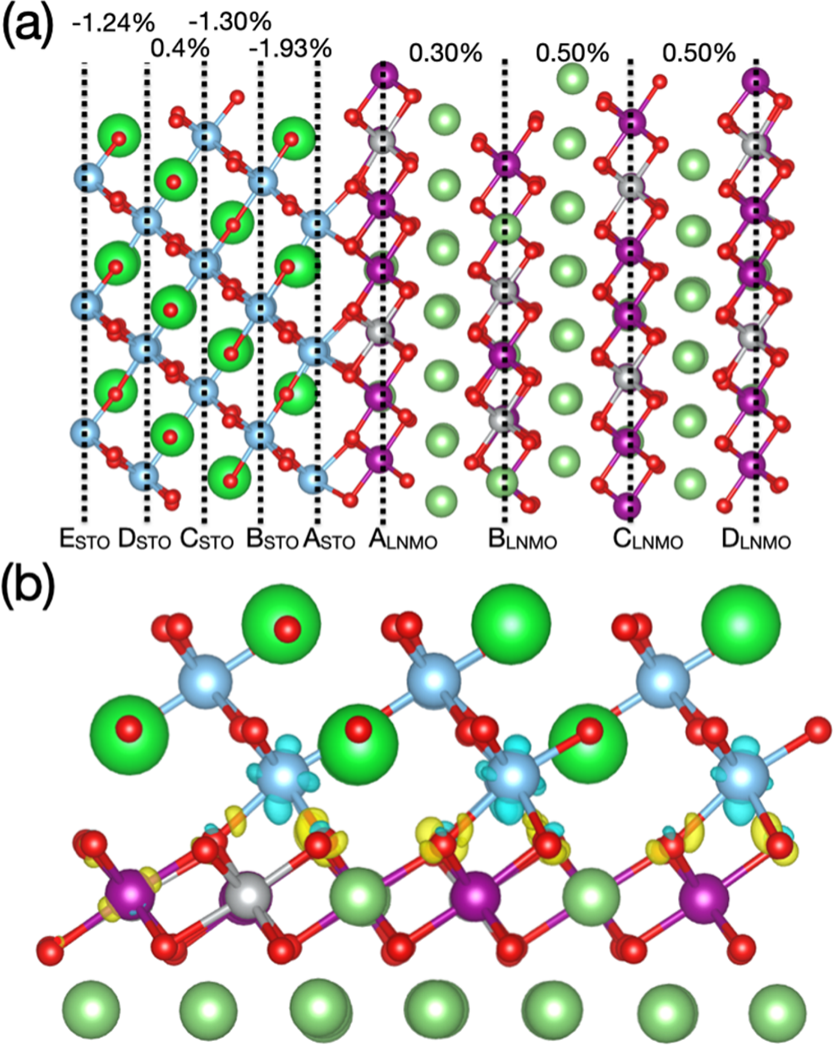
(a) Fully relaxed structure of the STO/LNMO interface system. Each layer is labeled according to its chemical composition and distance to the two-material interface. The percentages indicate the strain distortion in each layer along the direction perpendicular to the two-material interface as referred to their corresponding bulk system. (b) Charge density difference at the interface; electronic charge depletion and accumulation regions are represented by blue and yellow, respectively, and the isosurface value was set to 0.025 eV Å^–3^. The color code employed to represent the ions is the same as in Figure 1.

Figure 2 shows the fully relaxed STO/LNMO interface system obtained in our DFT simulations. Given the layered structure of LNMO in which nanosheets of Li and transition metal (TM)-oxygen ions alternate along the <001> direction, we started by choosing a rhombohedral LNMO <001> slab (for which we estimated a surface energy of +0.030 eV/Å^2^; eq 3). To minimize the interface strains and accompanying interface elastic energy, a STO slab with <111> orientation (with surface energy equal to +0.072 eV/Å^2^) was subsequently selected for building up the heterogeneous interface system (i.e., the STO <110> and STO <001> slabs cannot seamlessly match the 120° angle of rhombohedral LNMO in contrast to STO <111>; hence, they were disregarded in our modeling of a commensurate STO/LNMO supercell). The interface lattice mismatch between STO <111> and LNMO <001> prior to their epitaxy was 2.6%, which in the STO/LNMO interface system is entirely adopted by the STO slab given its condition of cathode coating (in other words, since in practice the thickness of the STO coating should be much smaller than that of the LNMO cathode, we assume that the structural distortions induced by the former on the latter can be safely neglected within the interface plane). The two slab terminations were selected so as to maximize the number of possible bonds between TM and oxygen ions at the STO/LNMO interface since these tend to be energetically very favorable (other possible surface terminations were not contemplated due their likely higher energy and our obvious computational limitations).

The lattice distortion for each layer along the direction perpendicular to the STO/LNMO interface as refereed to the corresponding bulk material was estimated as **η** ≡ (*a* – *a*_0_)/*a*_0_, where *a* and *a*_0_ represent the distance between consecutive layers in the interface and pure bulk systems, respectively. It was found that in the LNMO side **η** is quite small (i.e., 0.3% between A_LNMO_ and B_LNMO_ and 0.5% between C_LNMO_ and D_LNMO_), whereas in the STO side, it generally adopts very large and negative values (e.g., −1.93% for layers A_STO_ and B_STO_) (Figure 2a). The general out-of-plane contraction in the STO side and the general lack of out-of-plane distortion in the LNMO side can be easily understood in terms of elastic arguments typically employed for the description of thin films epitaxially grown on top of substrates. Since the epitaxial strain introduced by the interface in the STO (LNMO) block is high and tensile (null), the corresponding out-of-plane dimension significantly shrinks in an attempt to conserve its original volume (barely changes), thus minimizing the accompanying elastic energy.^41^

Epitaxial strain in a heterogeneous system requires the condition that atomic bonds bridging the distinct materials are formed at their interface. In the STO/LNMO system, we confirmed the presence of such bridging atomic bonds, involving Ti ions in the STO side and O ions in the LNMO side, through both charge density difference (CDD) (eq 2) and Bader charge analysis (Figure 2b). For instance, we found a significant accumulation (depletion) of negative charge in the LNMO (STO) layers lying closer to the boundary surface as compared to an isolated LNMO system. In particular, each interface O ion in the LNMO side received on average approximately 0.3 electrons from the neighboring Ti ions in the STO side. Moreover, we estimated the adhesion energy associated with the STO/LNMO interface and obtained −0.6 eV/Å^2^ (eq 4), which corroborates the presence of energetically favorable bonds uniting the LNMO <001> and STO <111> slabs.

**Figure 3:**
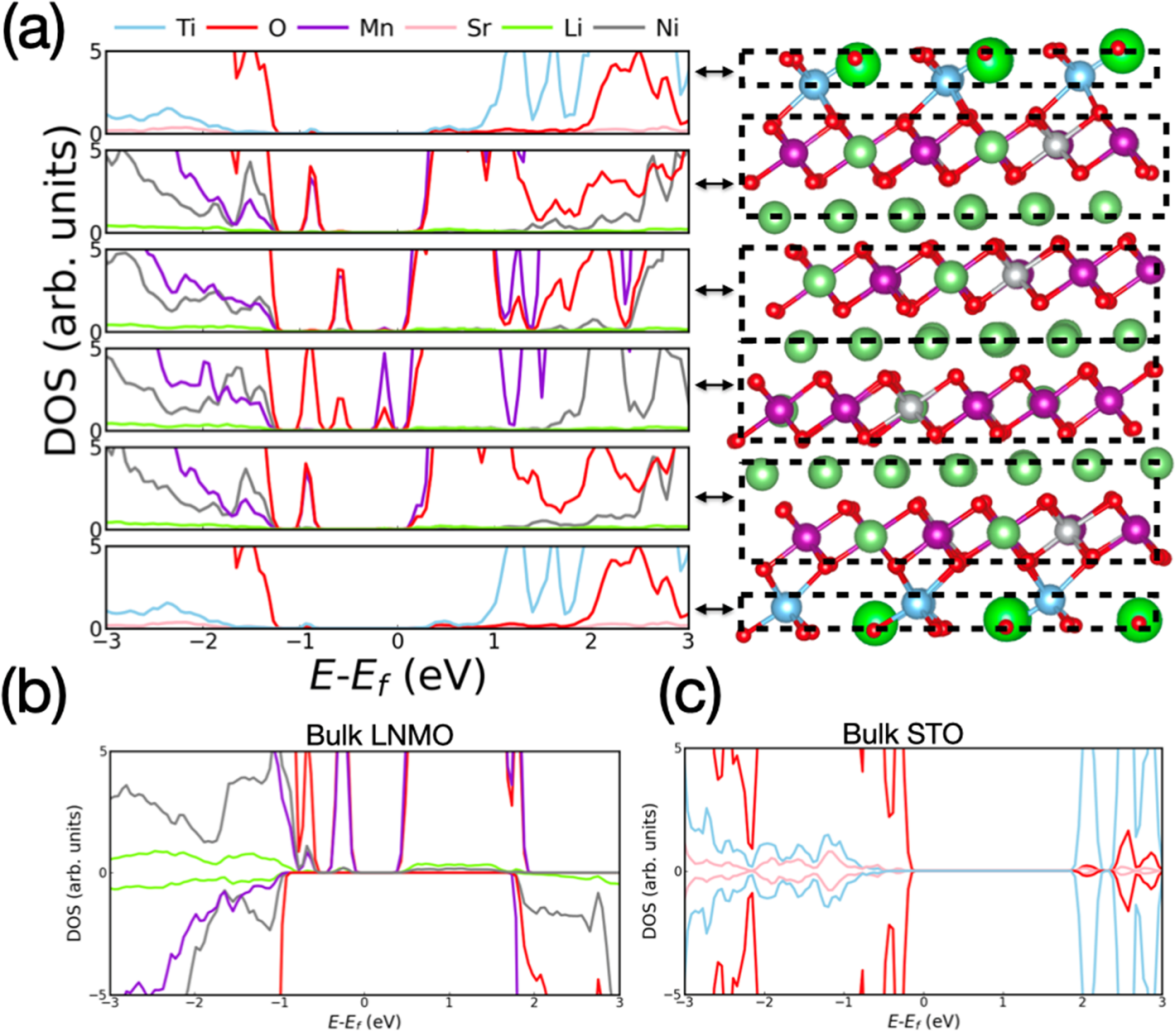
(a) Layered partial density of electronic states calculated for the STO/LNMO interface system; spin-down and spin-up states are summed up. Spin-polarized density of electronic states of bulk LNMO (b) and bulk STO (c).

To analyze in more detail the electronic band structure changes occurring in the STO/LNMO system, we calculated the corresponding layered partial density of electronic states (DOS; Figure 3a). All the energies in the DOS are shifted to zero with respect to the Fermi energy level (*E*_F_). It was observed that upon STO coating, the Fermi energy level in the LNMO layers closer to the interface shifts toward the bottom of the conduction band, thus rendering an *n*-type semiconductor (Figure 3a and Figure S4); on the contrary, bulk LNMO behaves like a *p*-type semiconductor since the corresponding *E*_F_ level lies closer to the top of the valence band (Figure 3b). Interestingly, electronic Mn and O states appear within the band gap of the interior LNMO layers (Figure 3a), thus appreciably reducing their energy band gap compared to that of bulk LNMO (which amounts to ∼0.24 eV; Figure 3b). The small, although non-zero, **η** strains between consecutive LNMO layers (Figure 2a) are likely to play a role in such a moderate band gap reduction. The band gap in the STO layers close to the interface was also found to decrease compared to the corresponding bulk value (i.e., ∼1.5 and ∼2.0 eV, respectively; Figure 3c), although it still remains large enough to block the possible diffusion of electrons toward the electrolyte. A transition from a *p*-type to an *n*-type semiconductor in moving from the bulk to the interface system, similar to that described for LNMO, is also observed in STO (Figure 3a–c).

### 3.2 Formation Energy and Impact of Oxygen Vacancies

Previous experimental and DFT-based simulation studies have shown that irreversible oxygen release at the surface of LRMO occurring during the charging process results in surface reconstruction to a rock-salt/spinel-like shell.^49,50^ In addition, the release of oxygen at the cathode surface may generate side reactions with the organic electrolyte, leading to capacity fading.^51,52^ Therefore, suppressing the release of oxygen at the cathode material surface is of critical importance for guaranteeing the stability and high energy capacity of LNMO.

**Figure 4:**
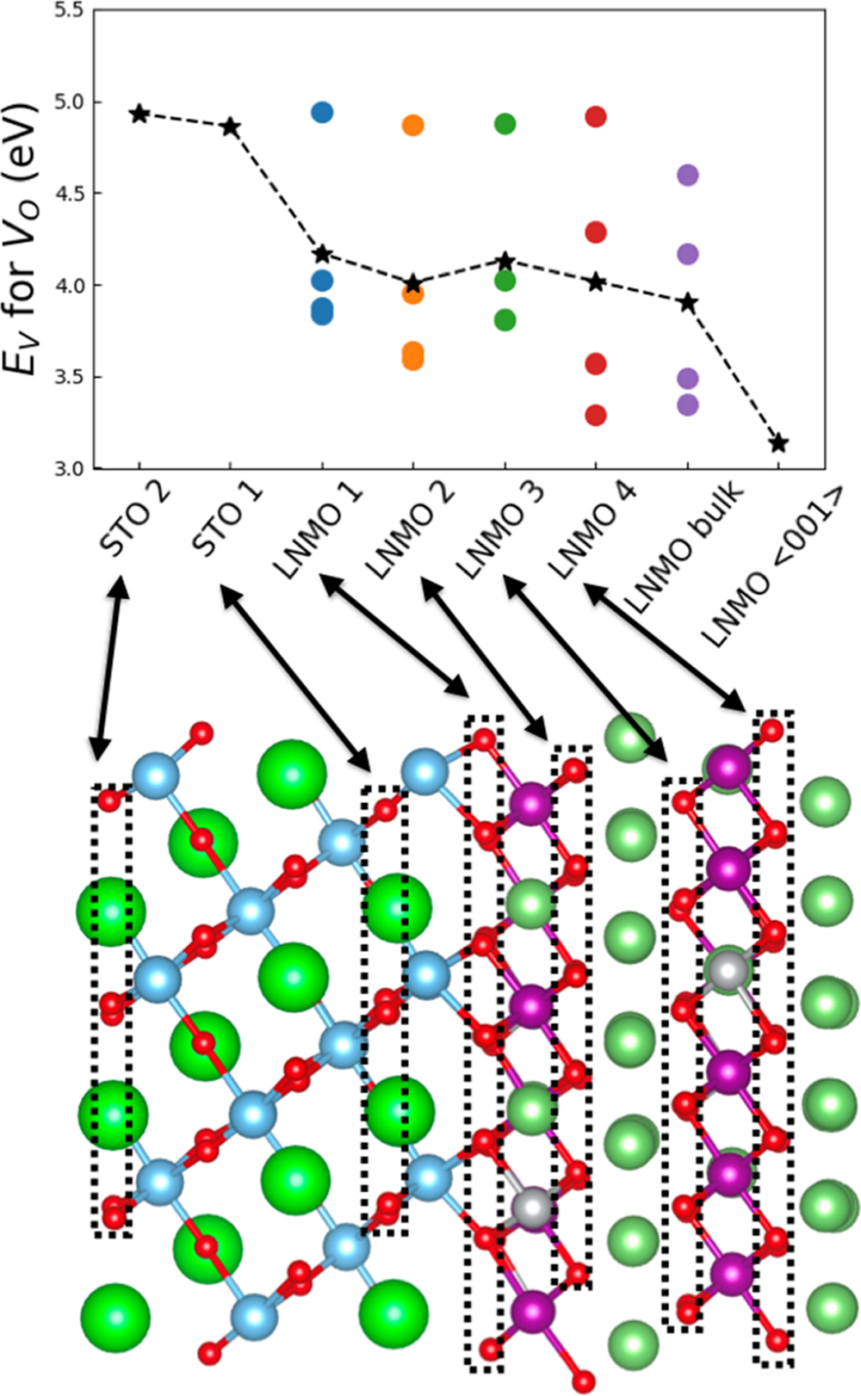
Oxygen vacancy formation energy calculated for different layers in the STO/LNMO interface system (indicated with dotted lines). Star markers are the average *E*_V_ values calculated in each layer. The *E*_V_ values estimated with analogous computational techniques for bulk LNMO and an exposed LNMO <001> surface are shown for comparison.

Motivated by these facts, we investigated the formation energy and impact of oxygen vacancies (O_V_) in the STO/LNMO system. To this end, a single oxygen atom was removed at a time from the STO and LNMO layers by considering all possible inequivalent positions (two in the first case and four in the latter; Figure S5). Figure 4 shows the formation energy of oxygen vacancies (*E*_V_; eq1) estimated for the STO and LNMO layers as a function of their distance to the interface (likely thermal effects were neglected^53^). Different points for a same layer represent the vacancy energies estimated considering different oxygen positions; the corresponding *E*_V_ average values are indicated with stars in Figure 4.

In the STO coating layers, *E*_V_ is largest (∼5 eV) and does not depend noticeably on the distance to the LNMO/STO interface, thus indicating that O_V_ are difficult to form in the protecting oxide perovskite coating. Near the STO/LNMO interface (i.e., “LNMO1” and “LNMO2” layers in Figure 4), *E*_V_ decreases by roughly 1 eV (∼4.0–4.2 eV) as compared to that in the oxide coating layer and is slightly higher than the one calculated for bulk LNMO (∼3.9 eV). For the inner LNMO layers (i.e., “LNMO3” and “LNMO4” layers in Figure 4), the estimated average *E*_V_ values are similar to those found close to the interface, thus suggesting that the small energy differences with respect to bulk are caused by the minute **η** strains appearing between consecutive LNMO layers (Figure 2a). Meanwhile, the average *E*_V_ calculated for an exposed LNMO <001> surface (i.e., with no oxide coating) amounts to ∼3.1 eV (Figure 4), which is noticeably smaller than those obtained for the STO/LNMO system due to the undercoordination of the oxygen ions in the surface. These results indicate that upon STO coating, oxygen loss from the LNMO surface can be partially prevented; hence, the undesirable layered to spinel/rock-salt phase transition might be inhibited in practice.^54^

**Figure 5:**
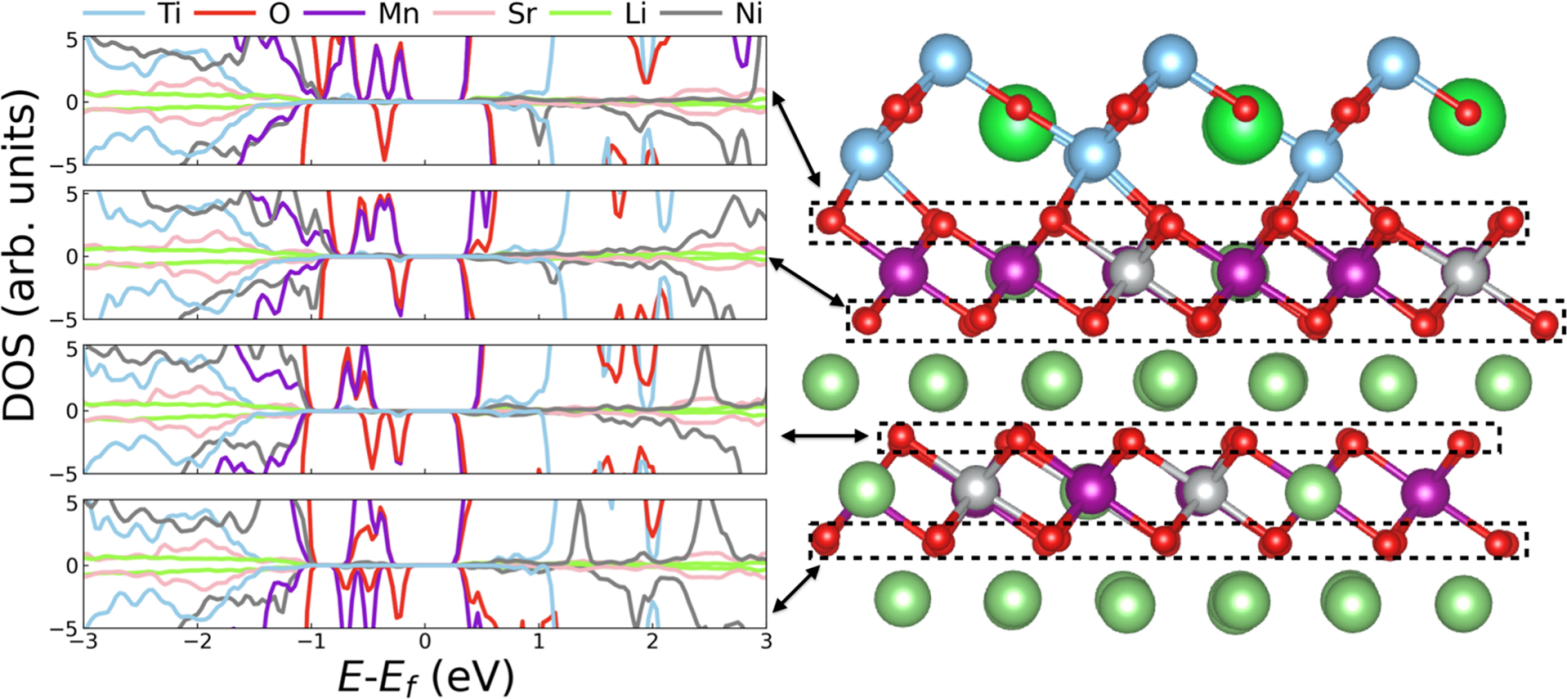
Layered partial density of electronic states calculated for different layers containing one oxygen vacancy in the STO/LNMO interface system. From top to bottom, plots correspond to the interface, second, third and fourth layers.

We analyzed the DOS of different LNMO layers containing oxygen vacancies in order to understand their impact on the electronic properties of the system (Figure 5). Interestingly, a band gap reemerges in the presence of O_V_ due to the disappearance of highly hybridized Mn 3*p* states and O 2*p* states near the bottom of the conduction band (Figure 5 and Figure S4). The O_V_-induced band gap amounts to ∼0.2 eV for all layers, thus indicating that the presence of oxygen vacancies may slightly hinder electronic conduction as compared to the analogous stoichiometric system (which shows no band gap). In addition, the presence of oxygen vacancies reverts the semiconducting character of the LNMO layers to *p*-type (i.e., like in the equivalent bulk system), and it increases the hybridization between the Mn 3*p* and O 2*p* orbitals at the interface layer. The CDD analysis performed on the system containing oxygen vacancies indicates that close to the STO/LNMO interface, the electrons left behind by the neutral O_V_ are transferred to the nearby Mn ions [Figure S6b]. Such charge transfers result in a hybridization between Mn 2*p* and O 2*p* electronic orbitals, which is stronger in one of the two spin channels at the interface than in the rest of interior LNMO layers (Figure 5).

### 3.3 Li Diffusion Properties

As mentioned in the Introduction, suitable cathode coating materials should be able to accommodate Li ions and allow for Li ion diffusion across them (complementary calculations and comments on the formation energy of Li vacancies are presented in the Supporting Information and Figure S7). NEB calculations were conducted to investigate the impact of coating on the Li diffusion properties of LNMO and STO. To mimic the coating scenario, LNMO and STO lattice distortions originated by the interface lattice mismatch between the two materials and the formation of interfacial atomic bonds were taken into consideration in our NEB calculations.

**Figure 6:**
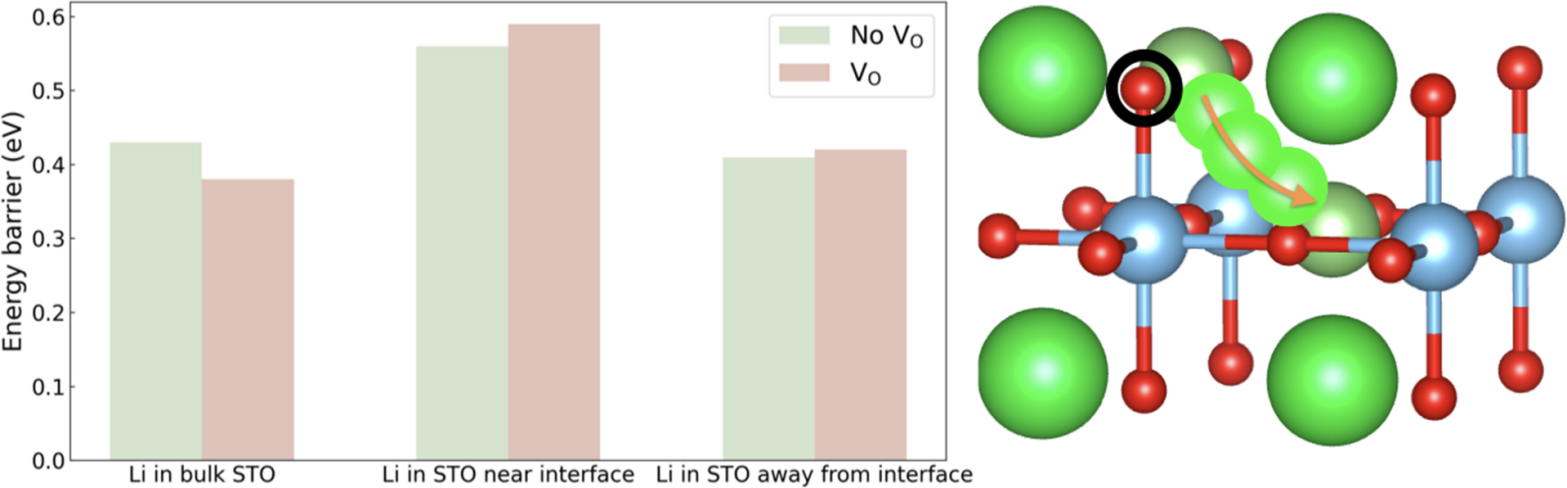
(Left) DFT-calculated NEB energy barrier (*E*_B_) for Li diffusion in stoichiometric and non-stoichiometric STO (cases “No V_O_” and “V_O_”, respectively). (Right) The Li diffusion path considered in the NEB calculations is represented with light green spheres (the orange arrow indicates the migration direction). The black circle indicates the location of the oxygen vacancy created along the Li diffusion pathway. Only the Li migration region of the system is shown for clarity.

Let us start with the analysis of the lithium diffusion properties of STO. Li ions were simulated as interstitials in the STO block. Since oxygen vacancies are a common type of point defects in STO thin films,^41^ we also simulated and analyzed the impact of V_O_ on Li diffusion (i.e., by considering an oxygen vacancy density of ∼2% in an 80-atom STO supercell). The STO lattice parameters between layers A_STO_ and B_STO_ (Figure 2a) were adopted to simulate Li transport near the STO/LNMO interface (Figure 6, case “Li in STO near interface”). Conversely, lattice parameters between layers C_STO_ and D_STO_ were adopted to simulate Li transport away from the STO/LNMO interface (Figure 6, case “Li in STO away from interface”). Meanwhile, the case of Li diffusion in fully relaxed bulk STO was also investigated (Figure 6, case “Li in bulk STO”) to provide meaningful comparisons with respect to the strained LNMO/STO interface systems. The lithium diffusion path considered in our NEB calculations is represented in Figure 6 along with the position of the created V_O_.

In the absence of oxygen vacancies, the estimated energy barrier for Li diffusion, *E*_B_, remains roughly equal to 0.4 eV in both the “Li in bulk STO” and “Li in STO away from interface” cases. Such a Li diffusion *E*_B_ similarity can be ascribed to the minor structural differences between the two implicated systems. In the “Li in STO near the interface” case, however, the Li diffusion energy barrier is found to increase up to 0.56 eV. This trend, which is also observed in the presence of V_O_, can be understood in terms of the sizeable structural distortions found in the LNMO/STO interface, which hinder coherent Li transport. Interestingly, the presence of V_O_ favors Li ion diffusion in the “bulk” case (i.e., *E*_B_ decreases by 0.05 eV), whereas it disfavors Li ion diffusion in the two considered interface cases. Specifically, *E*_B_ increases by 0.03 and 0.01 eV in the “Li in STO near interface” and “Li in STO away from interface” situations, respectively. The reason for this result seems to be related to the fact that the presence of V_O_ further promotes structural disorder near the LNMO/STO interface, which mildly reduces Li transport across it. Nevertheless, and importantly, the Li diffusion energy barriers calculated for STO in all the cases are similar in size to those estimated in previous DFT studies for good Li ion conductors,^55−58^ thus indicating that easy Li ion intercalation/deintercalation across the cathode coating is possible in practice.

**Figure 7:**
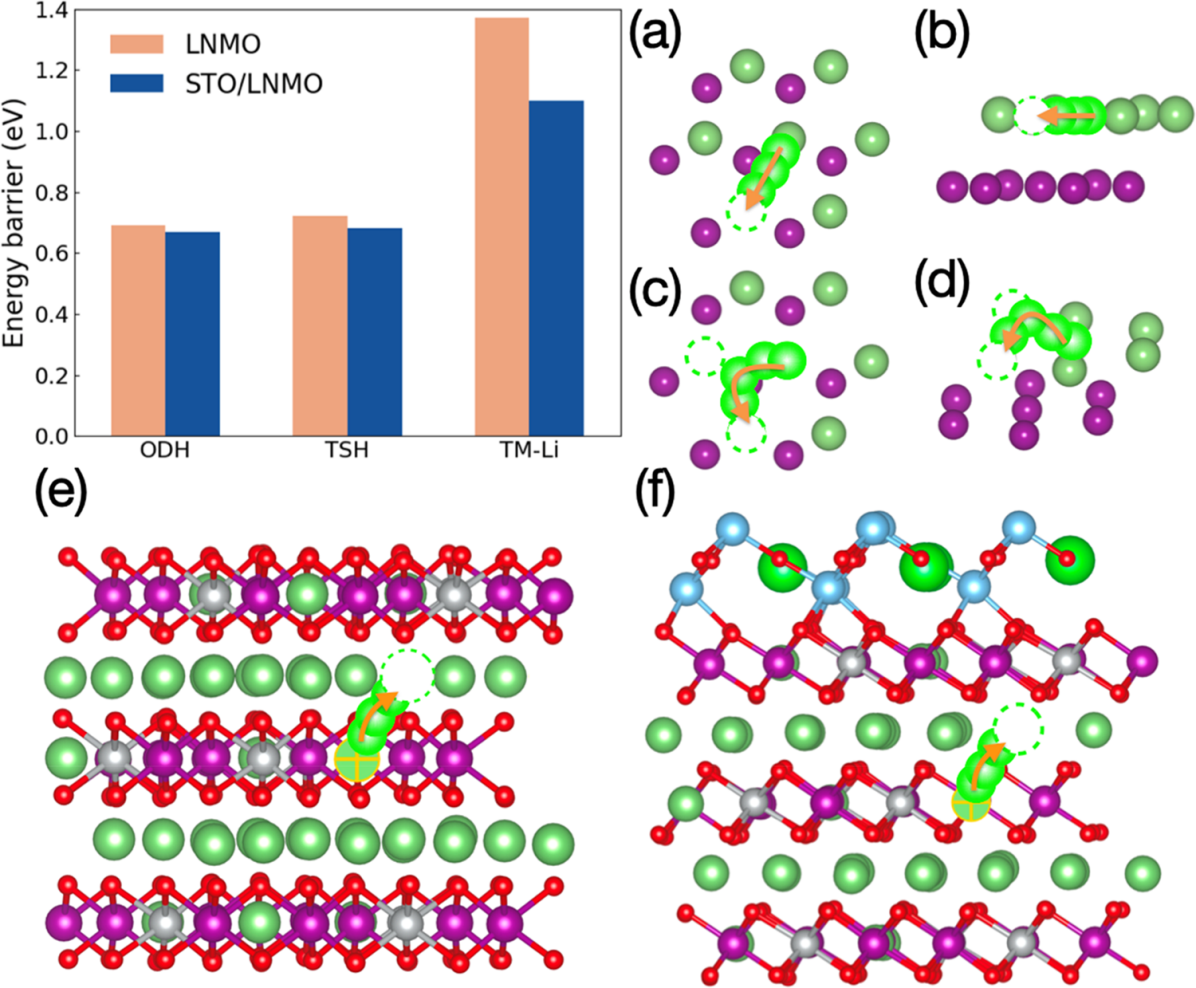
(Top left) Calculated DFT NEB energy barrier (*E*_B_) for Li diffusion in LNMO and the STO/LNMO interface system along two different diffusion paths (ODH and TSH); TM-Li represents Li diffusion along the <001> direction from a transition metal (TM) layer to a lithium layer. (a) Top view and (b) front view of the ODH diffusion path; (c) top view and (d) front view of the TSH diffusion path. Dark green and dark purple spheres indicate the Li and TM ions. (e) TM–Li diffusion path in bulk LNMO. (f) TMLi diffusion path close to the STO/LNMO interface. Light green spheres indicate NEB images for the Li atoms. Dotted circles indicate Li vacancies, and orange arrows indicate the direction of migrating ions. For the sake of clarity, many atoms have been removed from the crystal structure plots in (a) to (d).

Next, we comment on the DFT results obtained for the Li ion diffusion properties of LNMO. In analogy to STO, two different cases were considered: (i) bulk LNMO, which can be regarded as the LNMO cathode away from the interface, and (ii) STO/LNMO, which mimics the STO interfacial region in the LNMO cathode. There are several possible diffusion pathways to be considered in layered oxide compounds; however, in a previous DFT work by Van der ven *et al.*, the two most likely pathways were already identified. Those two most likely Li diffusion pathways are shown in Figure 7 and involve oxygen dumbbell hops (ODH) and tetrahedral site hops (TSH). In the ODH case, the Li ion migrates along the shortest path connecting the initial Li site and the oxygen vacancy site (Figure 7a,b). With regard to the TSH pathway, the lithium ion migrates along a curved path passing through a tetrahedral site surrounded by two Li vacancies (Figure 7c,d). This is the mechanism by which the hopping Li ion ends up forming a divacancy.^59^

In the STO/LNMO system, it is observed that the *E*_B_ decreases by 0.02 and 0.04 eV for the ODH and TSH pathways, respectively, as compared to bulk LNMO. These outcomes suggest that upon STO coating, the Li ionic conductivity properties of LNMO will not be deteriorated but actually somewhat improved. The main cause of the *E*_B_ reduction found in the STO/LNMO system is the structural elongation that is attained along the axis perpendicular to the interface (Figure 2a): the distance between two consecutive Li layers increases by ∼0.3%, which facilitates the passage of Li ions through both the ODH and TSH pathways.^45^ The majority of the lattice expansion is accommodated by the Li layers since the transition metal (TM)–O bonds are stiffer than the Li–O bonds, thus providing additional free space for the ionic motion^60^ (it is worth mentioning that in a previous DFT study it has been shown that a 4% increase in the *c* lattice parameter of LiCoO_2_ can change *E*_B_ by more than 200%;^60^ hence, Li diffusion is very sensitive to the structural parameters of the cathode material). We note that in the STO/LNMO system Li diffusion is slightly more favorable through the ODH than the TSH pathway since the accompanying *E*_B_ is smallest. This outcome is different from the results reported for other more delithiated materials in which the TSH path seems to be preferred over the ODH.^44^ However, in our STO/LNMO system, since the concentration of Li vacancy is quite low (i.e., 1.6% for a single Li vacancy), the ODH path is likely to dominate the Li diffusion.

Moreover, the energy barriers involved in Li diffusion across inner LNMO layers (i.e., along the <001> direction) have been also calculated (Figure 7a–e,f) (we note that the initial and final positions for a lithium ion moving from a TM layer to a Li layer are not equivalent and that the migration energy barriers reported here for interlayer diffusion correspond to the TM–Li path sketched in Figure 7). Specifically, the *E*_B_ involved in lithium interlayer migration roughly amounts to 1.4 eV in bulk LNMO, which is about 2 times larger than the energy barriers estimated for the ODH and TSH hopping paths. Nevertheless, upon STO coating, this energy barrier is reduced by ∼0.3 eV (Figure 7a). The cause of such an *E*_B_ decrease may be understood again in terms of the small interlayer expansion that LNMO experiences upon STO coating (Figure 2a), which facilitates the passage of lithium ions across them.

**Figure 8:**
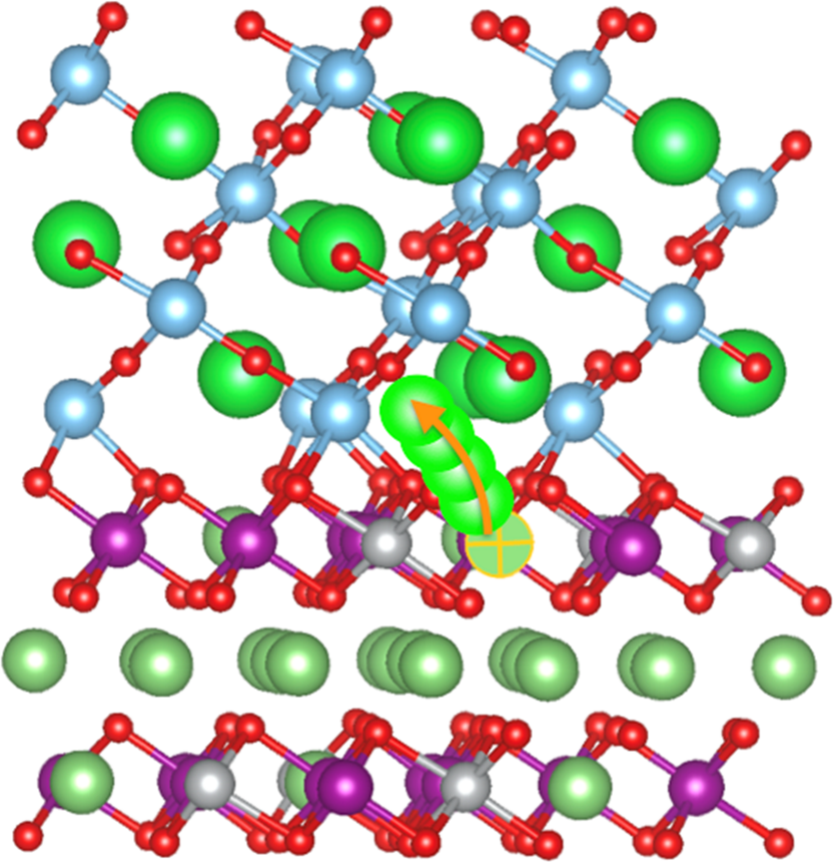
Analyzed Li migration pathway across the STO/LNMO interface. Light green spheres represent the NEB images of the diffusing Li atom, and the orange arrow represents its path direction.

Finally, we conducted additional NEB calculations to estimate the energy barrier involved in Li migration across the STO/LNMO interface (Figure 8). As it was expected from the outcomes of a previous similar first-principles study,^32^ the *E*_B_ calculated in this case is appreciably larger than those found in the previous STO and LNMO systems, and it roughly amounts to 2.0 eV. This result implies that in practice, coating the LNMO cathode with STO may result in the appearance of a non-negligible interfacial resistance that to a certain extent may hinder the diffusion of Li ions across the STO/LNMO junction under normal battery operation conditions. Nonetheless, the existence of other possible Li diffusion paths involving smaller energy barriers should not be discarded on the basis of our NEB calculations.^42^

We note that our ionic transport conclusions obtained for the fully lithiated STO/LNMO system may change upon variation of the Li content in the cathode material. Nevertheless, due to obvious computational limitations, we leave the detailed study of delithiated STO/LNMO systems to future works.

## 4 Conclusions

Simulation techniques based on density functional theory were employed to assess the potential of STO, an archetypal and highly versatile oxide perovskite, as a protective coating for LRMO-based cathode materials in electrochemical lithium-ion batteries. Our theoretical DFT results obtained for a detailed atomistic STO/LNMO interface system show that the LNMO band gap near the coating boundary is reduced due to the presence of Mn and O mid-gap states, which enhances electronic transport within the LNMO cathode. By analyzing the formation energy of oxygen vacancies in different transition-metal environments, we concluded that upon STO coating, LNMO oxygen loss becomes less likely especially near the interface, a result that confirms the usefulness of protective oxide perovskite layers in preventing unwanted LRMO spinel-to-rock-salt phase transformations. Moreover, NEB calculations reveal that Li ions can easily diffuse through STO and that upon oxide perovskite coating, lithium transport in LNMO is enhanced due to the induced structural distortions that tend to slightly increase the spacing between consecutive oxide layers. Additionally, NEB calculation shows the decreased migration energy barrier for interlayer hopping, proving that it is highly likely Li migrating from LNMO to STO. Therefore, the present computational DFT study concludes that STO, and probably also other analogous oxide perovskite materials like LaAlO_3_ and SrMnO_3_ that are neither polar, may be used in practice as efficient cathode LRMO coating materials. We hope that this work will motivate experimental efforts toward the implementation of protective and functional oxide perovskite coatings in high-energy density and capacity ion batteries, thus making a potential impact in the technological fields of portable electronic devices and transportation.

## Supporting Information Available

Additional simulation details on the analyzed materials including lattice parameters, macroscopic average electrostatic potentials, structural and electronic properties, and the formation energy of Li vacancies in the STO/LNMO system (PDF)
