# Supplementary material for: Tuning the Electronic, Ion Transport, and Stability Properties of Li-rich Manganese-based Oxide Materials with Oxide Perovskite Coatings: A First-Principles Computational Study
Source: ACS Appl Mater Interfaces. 2022 Aug 5;14(32):37009–18. doi: 10.1021/acsami.2c07560 (PMC9389528; doi:10.1021/acsami.2c07560)
Supplement: Supplementary file 1 — am2c07560_si_001.pdf [file am2c07560_si_001.pdf]

Supporting Information

Tuning the Electronic, Ion Transport and Stability Properties of Li-rich Manganese Based Oxides Materials with Oxide Perovskite Coatings: A First-Principles Computational Study

Zizhen Zhou,[a, b, c]* Dewei Chu,[a] Bo Gao,[c] Toshiyuki Momma,[b] Yoshitaka Tateyama[b, c] and Claudio Cazorla[d]**

a. School of Materials Science and Engineering, UNSW Australia, Sydney NSW 2052, Australia
b. Graduate School of Advanced Science and Engineering, Waseda University, 3-4-1, Okubo, Shinjuku-ku, Tokyo 169-8555, Japan
c. Center for Green Research on Energy and Environmental Materials (GREEN) and International Center for Materials Nanoarchitectonics (MANA), National Institute for Materials Science (NIMS), 1-1 Namiki, Tsukuba, Ibaraki 305-0044, Japan
d. Departament de Física, Universitat Politècnica de Catalunya, Campus Nord B4-B5, E-08034 Barcelona, Spain

* zizhen_zhou@toki.waseda.jp

** claudio.cazorla@upc.edu

Table S1: Simulation lattice parameters for bulk STO[1] and bulk LNMO[2] and the combined STO/LNMO interface supercell. The SrTiO3 <110> and SrTiO3 <001> orientations were disregarded in the construction of a commensurate STO/LNMO supercell due to the impossibility of matching the γ=120º angle of the rhombohedral LNMO <001> slab (γ being the angle formed by the supercell lattice vectors **u** and **v** defining the interface plane).

	a (Å)	b (Å)	c (Å)	Reference (Å)
**$SrTiO_3$ (bulk)**	3.95	3.95	3.95	3.905[1]
**$Li_{1.2}Ni_{0.2}Mn_{0.6}O_2$ (bulk)**	2.90		14.38	a = 2.91<br>c = 14.26[2]
	**u (Å)**	**v (Å)**	**γ (º)**	**Lattice parameter mismatch**
**$SrTiO_3$ <111>**	11.18	11.18	120	
**$Li_{1.2}Ni_{0.2}Mn_{0.6}O_2$ <001>**	11.47	11.47	120	2.6%

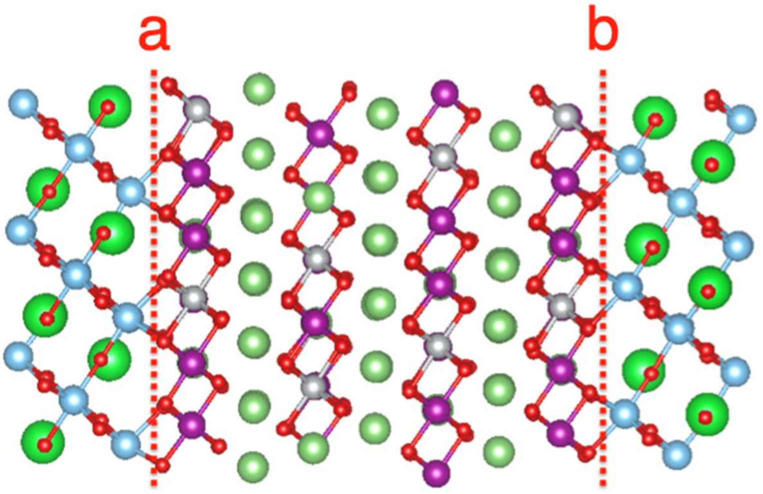

Figure S1: Sketch of the STO/LNMO interface system. Dotted lines indicate the location of two interfaces in the supercell. The simulation supercell is practically symmetric along the direction perpendicular to the two-materials interface. Li, Mn, Ni, Sr, Ti and O atoms are represented with dark green, purple, grey, light green, blue and red spheres, respectively.

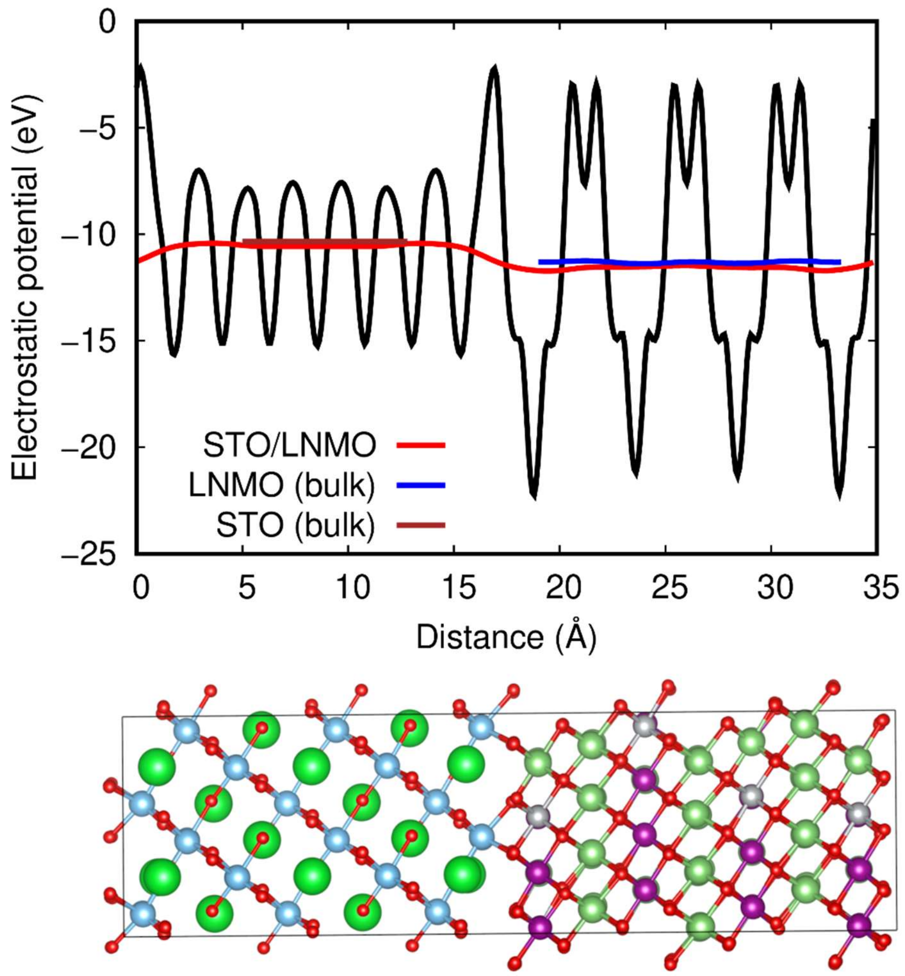

Figure S2: Macroscopic average electrostatic potential estimated for the STO/LNMO interface system along the direction perpendicular to the two-materials interface. The good correspondence found between the macroscopic average electrostatic potentials of the heterogenous system and bulk STO and bulk LNMO indicates good convergence of our DFT results with respect to the thickness of the slabs.

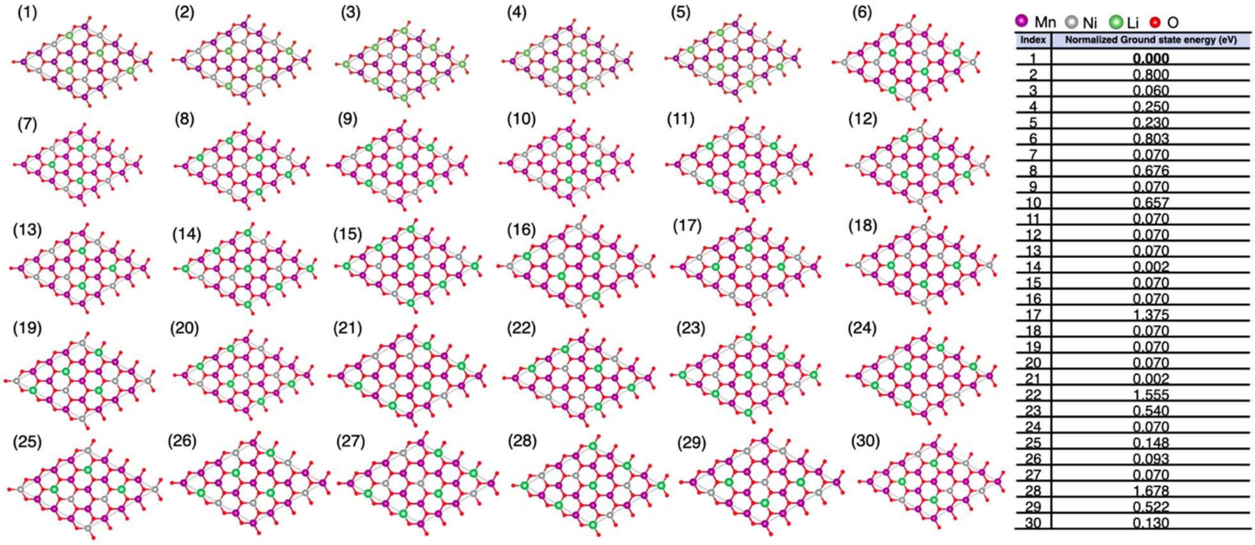

Index	Normalized Ground state energy (eV)
1	**0.000**
2	0.800
3	0.060
4	0.250
5	0.230
6	0.803
7	0.070
8	0.676
9	0.070
10	0.657
11	0.070
12	0.070
13	0.070
14	0.002
15	0.070
16	0.070
17	1.375
18	0.070
19	0.070
20	0.070
21	0.002
22	1.555
23	0.540
24	0.070
25	0.148
26	0.093
27	0.070
28	1.678
29	0.522
30	0.130

Figure S3: (1)-(30) show the first 30 LNMO structures generated by Pymatgen and that have been considered in our preliminary configurational analysis; the table shows the corresponding DFT energies. All the energies are referred to the most stable structure according to Pymatgen and DFT, namely, (1). The local Mn-Ni-Li arrangements have been geometrically optimized in all the cases.

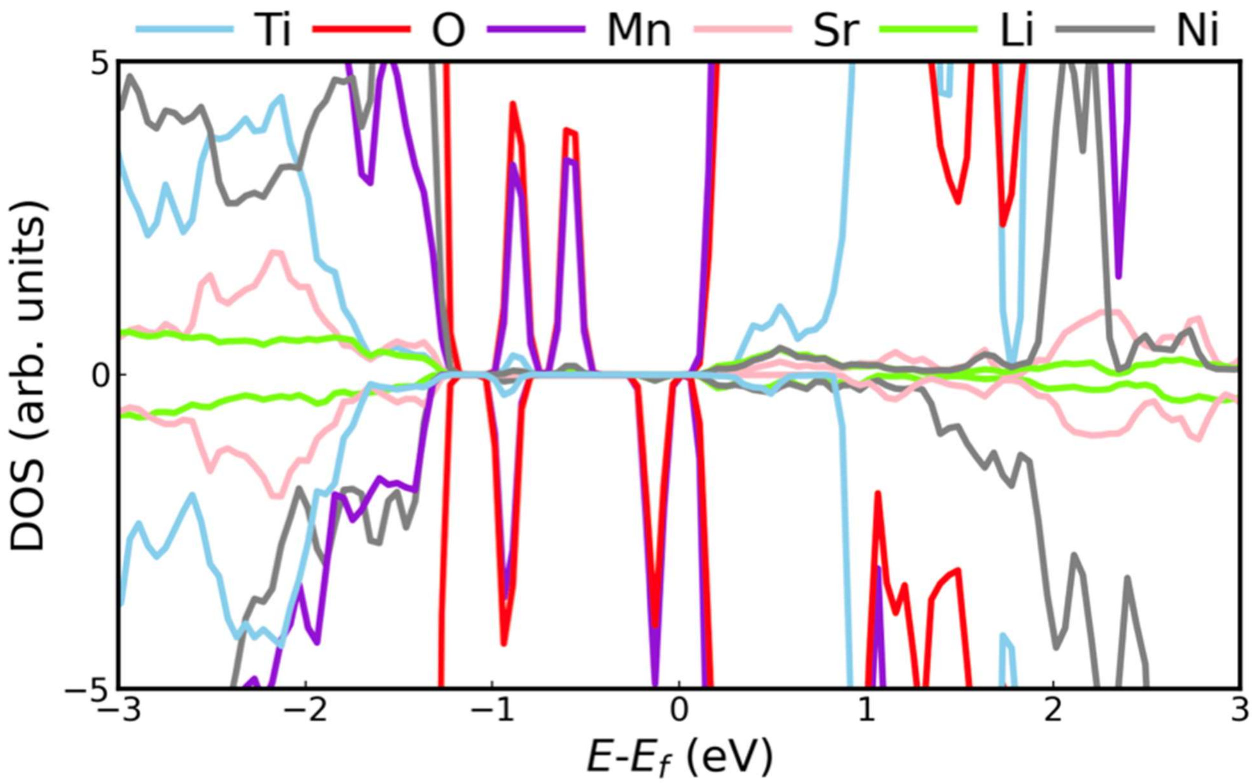

Figure S4: Atomic projected density of state of STO/LNMO interface structure.

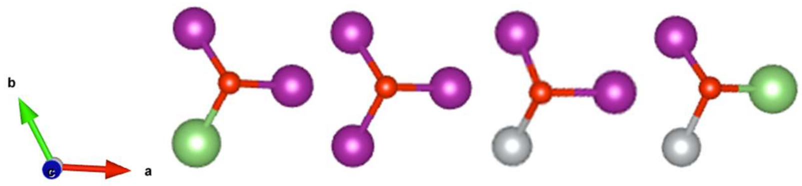

Figure S5: Four types of oxygen configurations in LNMO. The color code is the same that in Figure S1.

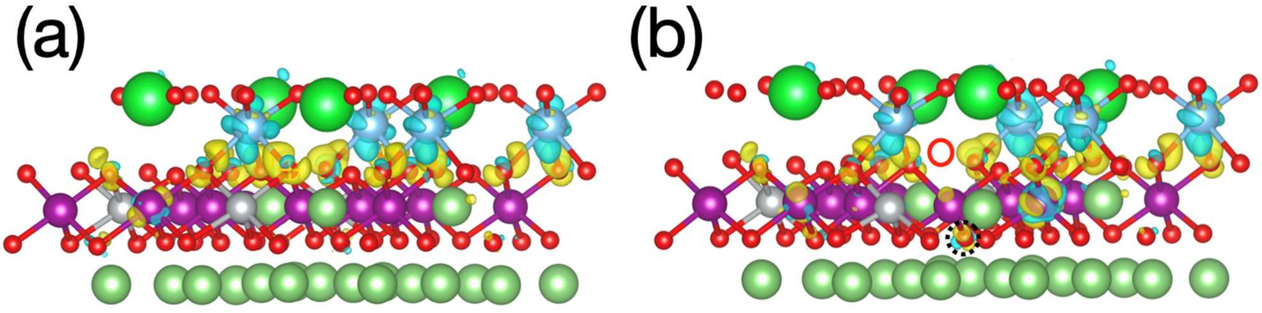

Figure S6: charge density distribution comparison between (a) stoichiometric and (b) O_V containing LNMO/STO systems. Cross mark in (a) indicates the removed oxygen. Black dotted circle in (b) indicates a newly formed region of charge accumulation (as compared to the stoichiometric system). Red circle indicates the site of oxygen vacancy. The color code is the same that in Figure S1.

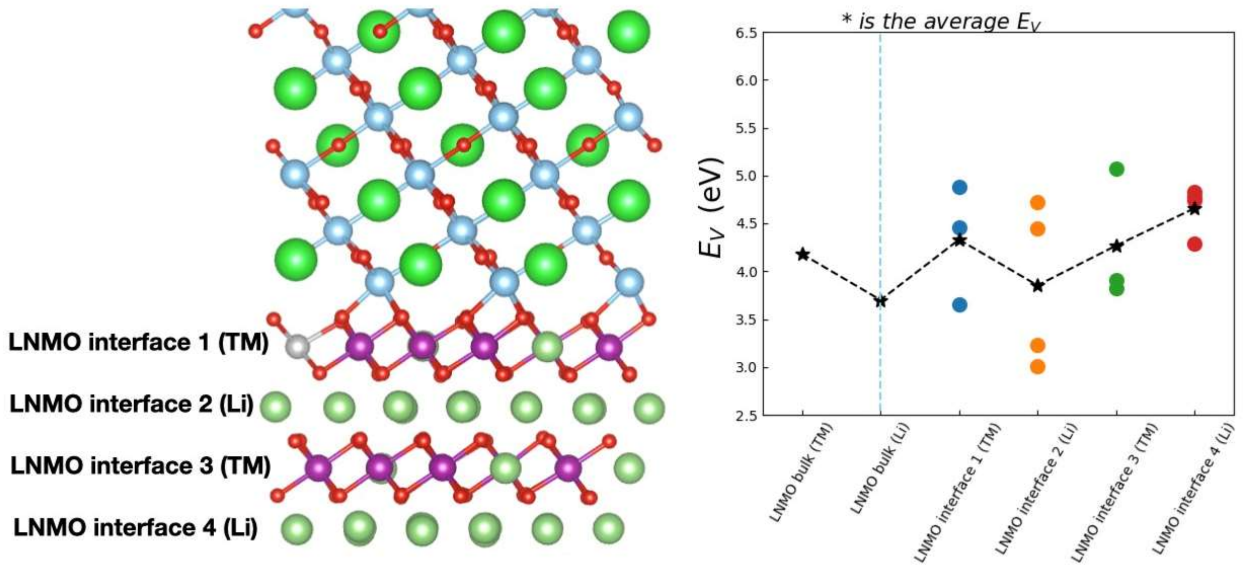

Figure S7: Calculated E_V for Li atoms at different positions in "TM" and "Li" layers; star markers represent average values for each layer. For comparison purposes, the analogous E_V values estimated for bulk LNMO are shown. The color code is the same that in Figure S1.

Supporting Discussion: Li vacancy formation energy

The formation energy of Li vacancies [E_V, Eq.(1) in the "Computational Method" section] in the LNMO region of the interface LNMO/STO model was estimated as a function of the distance to the materials boundary. Figure S7 shows the selected LNMO layer in which single Li vacancies were created. Three different Li ions in the TM layers were removed separately to explore all possible types of Li vacancy, whereas in the pure Li layers there are four different types of Li vacancies (Figure S7). It can be appreciated that in average there is not much E_V variation in the TM layers as compared to the corresponding bulk LNMO case. Interestingly, Li vacancies created in Li layers show a different behavior. In particular, we found a larger E_V dispersion near the interface ['LNMO interface 2 (Li)'], in which E_V varies in the range of 3.0-4.7 eV. It is shown that the lowest E_V value corresponds to the first Li layer near the interface. This result indicates that upon STO coating there is a dramatic change in the local environment of Li ions near the interface, which facilitates their removal [i.e., the E_V results obtained for the 'LNMO interface 4 (Li)' layer render a smaller variance but larger average value of ~4.7 eV].

References :

(1) Janotti, A.; Jalan, B.; Stemmer, S.; Van de Walle, C. G. Effects of doping on the lattice parameter of $SrTiO_3$. *Applied Physics Letters* **2012**, *100* (26), 262104.
(2) Li, X.; Qiao, Y.; Guo, S.; Xu, Z.; Zhu, H.; Zhang, X.; Yuan, Y.; He, P.; Ishida, M.; Zhou, H. Direct Visualization of the Reversible O^{2-}/O^{-} Redox Process in Li-Rich Cathode Materials. *Advanced Materials* **2018**, *30* (14), 1705197.
